# Some Methods of Utilising Hospital Waste

**Published:** 1906-02-10

**Authors:** F. A. Washburne

**Affiliations:** Assistant Medical Superintendent, Massachusetts General Hospital


### HOSPITAL ADMINISTRATION.

### CONSTRUCTION AND ECONOMICS.

### SOME METHODS OF UTILISING HOSPITAL WASTE.

By Dr. F. A. Washburne, Assistant Medical Superintendent, Massachusetts General Hospital.

Two main principles are involved in reducing hospital expenditure: (1) Using less new material; (2) utilising waste material. In this paper I will dwell principally upon some of the methods which may be employed in carrying-out the second of these two principles—namely, utilising material which has formerly gone to waste.

The item of absorbent gauze is a large one in every general hospital. At the Massachusetts General Hospital in 1902 it amounted to $6,079.81 (£1,220), in 1903 to $5,959.37 (£1,120). Up to one year ago at this hospital it had been customary to destroy all gauze which had been soiled by blood or pus, and I believe that this was the rule at all hospitals, although some had made an attempt to recover the gauze from their clean cases. In October 1904, with the consent of the Visiting Staff, a process of washing and sterilising all gauze was adopted, and its success has exceeded our expectations.

#### The Saving Effected—£600 a Year.

In the first eight months of 1904 we used over 140 miles of new gauze 3 feet wide. In the first eight months of 1905 we used 51 miles. The new gauze used in the first eight months of 1904 would have stretched from Boston nearly to New Haven, Conn., while in a corresponding period of 1905 it would have reached only five miles beyond Providence, R.I. Money actually expended for gauze during the first eight months of the following years was: 1900, $3,774.01 (£755); 1901, $4,275.91 (£855); 1902, $3,872.61 (£775); 1903, $4,029.22 (£806); 1904, $4,366.52 (£873); 1905, $1,253.40 (£251). That shows a saving in 1905 of about $3,000.00 or £600 in eight months over the cost in 1903 and 1904.

#### The System Explained.

The method employed is as follows: All gauze and bandages from ward dressings, amphitheatre, and out-patient department and operating rooms is collected in paper bags and taken to the laundry. It is transferred from these paper-bags to open-work bags made of cord, these bags being only half filled. The gauze is kept in these bags throughout the rest of the process of washing and the laundry sterilisation. It is put in soak overnight in cold water which is changed several times. The following morning it is put into an iron washer capable of resisting steam pressure up to ten pounds. It is first washed in cold water until the water runs perfectly clear. The gauze is then washed with warm water, soap, and soda. After this washing it is rinsed in hot water. After the rinsing, enough hot water is turned into the washer to cover the bags of gauze as they lie on the bottom of the washer. Steam is then turned on to a pressure of ten pounds. A self-registering thermometer placed in the gauze twice showed a temperature of 239 and 240 degrees. The thermometer which registers on the outside of the washer showed a temperature of 236 degrees at pressure of ten pounds. This temperature is maintained for one half-hour. During all this process the washer is moving with a to-and-fro motion which continually agitates the gauze and presents all parts of it to the motion of the water and steam.

The gauze is then put into the extractor and when dry is sent to the out-patient department where it is overhauled under the direction of an intelligent maid. It is untangled and straightened, and the maid is instructed to throw out any piece of gauze which is stained or has anything adherent to it. The gauze is again handled by nurses or maids, when it is cut and placed in packages for the last sterilisation. These women, also, have instruction to look the gauze over carefully for any stained piece or any piece with foreign matter adherent. The final sterilisation is then done at a temperature of 250 degrees F. with a pressure of 15 pounds in the sterilising room.

#### The Actual Results in Materials.

It is found that the washed gauze is softer and more absorbent than the new gauze. Repeated tests conducted in the pathological laboratory have shown the safety of the process. The overhauling and straightening of the washed gauze involves a very considerable amount of labour, and if it were necessary to hire additional people to do the work the saving would be much less. At the Massachusetts General Hospital we utilise labour that would otherwise go to waste. In our large out-patient department we must have a certain force of messenger-boys and maids when the clinic is at its largest and at the busiest hours of the day. Much of the time all this force is not required for running the clinics or cleaning the buildings, and their time is utilised on the gauze.

#### Small Pieces and Waste Utilised.

So much for the gauze which is recovered and utilised again as gauze. There is a part which is in too small pieces or is too badly tangled to be worth straightening. This material is run through a rag-picker and becomes a very light and absorbent lint which is sterilised and used in dressings where absorbent cotton or oakum is ordinarily used. It is also used in the boiler-house in the place of waste for wiping around the engines. Another part of the gauze is thrown out because it is stained with chemicals. These pieces are utilised by the house-cleaning force. This process, therefore, means not only less gauze bought, but less absorbent cotton, less oakum, less waste for the engine-room. All pieces of compress cloth, many of which have been thrown away in the past, are washed and sterilised by this same process, used over again if large enough, run through the picker if small.

A Further Check upon Waste.

Packages which are sent to the boiler-house to be burned are overhauled and inspected at frequent and irregular intervals, and if it is found that material which should be saved is being wasted, the responsible head nurse is called to account. Safety-pins, rubber dam, knives, forks, and spoons are recovered from this inspection. I believe that it will pay to have a systematic inspection by a careful employé of everything brought from any part of the hospital to be destroyed or thrown away. Such a man cannot fail to more than earn his pay and board, nor would his inspection work take the whole of his time.

Soap Making.

About one year ago the Massachusetts General Hospital started to make its own soap. I have endeavoured to make a comparison of the cost to the hospital of buying soap for the seven months, February to August, 1904, and making it for the seven months, February to August, 1905. We formerly used in the laundry a soap chip for which we paid about 4¾ cents (2½d.) per pound. We used a common yellow bar soap for which we paid 5 to 5½ cents (say 2½d.) per pound. Savogran for the floors, etc., at 6 cents (3d.) per pound, and soft soap for ward use at 3½ cents (1¾d). All these have been nearly or quite replaced by our home-made soft soap, which we find answers all these purposes in a satisfactory manner. We still use a small amount of savogran on the white tile floors of our surgical building, and a little bar yellow soap here and there. This latter we shall make ourselves as soon as our stock on hand is used up. But soft soap practically replaces everything except sand soap, toilet soap, soap polish, etc., which we buy as before.

In the seven months of 1904 under consideration, we paid $535.26 more for savogran than in the seven months of 1905, $108.48 more for yellow bar soap, $24.99 more for soft soap, and $300.65 more for soap chips. A total of $969.38 more paid for soap in seven months of 1904 than in the seven months of 1905. This $969.38 represents the saving effected by our home soap making in the period named.

To counterbalance this we received in the seven months of 1905 $255.15 less for grease. Soap making takes about a third of the time of one man, estimated at $177.25 for seven months. Potash cost $293.91. Steam used in cooking soap estimated at $5 per month, $35. A total saving of $268.07 in seven months.

We have less trouble with our washers in the laundry because of the larger amount of free alkali in the soap made by our recipe, which is practically the same as that used at the McLean Hospital and the State Hospital. It is as follows:—

Dissolve 22 lb. of potash in 6 nine-quart pails of water, add 34 lb. of grease and boil very slowly 6 hours. Then fill a 100 gallon tank half full of water and let it come to a boil again. When it becomes stringy turn off the steam and fill the tank with water.

The soap has been tested by the hospital apothecary and is found to correspond to the maximum of free alkali allowed by the 1900 United States Pharmacopœia. Various fabrics have been allowed to soak in this soap for several days and have shown no detriment. The practical test of a year's use shows no injury to clothing.

